# Feasibility of Using a Commercial Fitness Tracker as an Adjunct to Family-Based Weight Management Treatment: Pilot Randomized Trial

**DOI:** 10.2196/10523

**Published:** 2018-11-27

**Authors:** Thao-Ly Tam Phan, Nadia Barnini, Sherlly Xie, Angelica Martinez, Lauren Falini, Atiera Abatemarco, Maura Waldron, Jane M Benton, Steve Frankenberry, Cassandra Coleman, Linhda Nguyen, Cindy Bo, George A Datto, Lloyd N Werk

**Affiliations:** 1 Center for Healthcare Delivery Science Nemours Children's Health System Wilmington, DE United States; 2 Department of Pediatrics Sidney Kimmel Medical College Thomas Jefferson University Philadelphia, PA United States; 3 Division of Weight Management Nemours Children's Health System Wilmington, DE United States; 4 Cardiac Center Nemours Children's Hospital Orlando, FL United States; 5 Biostatistics Core Department of Biomedical Research Nemours Children's Health System Wilmington, DE United States; 6 Division of General Academic Pediatrics Nemours Children's Hospital Orlando, FL United States; 7 Department of Kinesiology West Chester University West Chester, PA United States; 8 Strategy and Business Development Nemours Children's Health System Wilmington, DE United States; 9 College of Medicine University of Central Florida Orlando, FL United States

**Keywords:** fitness trackers, pediatric obesity, health behavior, accelerometry

## Abstract

**Background:**

Fitness trackers can engage users through automated self-monitoring of physical activity. Studies evaluating the utility of fitness trackers are limited among adolescents, who are often difficult to engage in weight management treatment and are heavy technology users.

**Objective:**

We conducted a pilot randomized trial to describe the impact of providing adolescents and caregivers with fitness trackers as an adjunct to treatment in a tertiary care weight management clinic on adolescent fitness tracker satisfaction, fitness tracker utilization patterns, and physical activity levels.

**Methods:**

Adolescents were randomized to 1 of 2 groups (adolescent or dyad) at their initial weight management clinic visit. Adolescents received a fitness tracker and counseling around activity data in addition to standard treatment. A caregiver of adolescents in the dyad group also received a fitness tracker. Satisfaction with the fitness tracker, fitness tracker utilization patterns, and physical activity patterns were evaluated over 3 months.

**Results:**

A total of 88 adolescents were enrolled, with 69% (61/88) being female, 36% (32/88) black, 23% (20/88) Hispanic, and 63% (55/88) with severe obesity. Most adolescents reported that the fitness tracker was helping them meet their healthy lifestyle goals (69%) and be more motivated to achieve a healthy weight (66%). Despite this, 68% discontinued use of the fitness tracker by the end of the study. There were no significant differences between the adolescent and the dyad group in outcomes, but adolescents in the dyad group were 12.2 times more likely to discontinue using their fitness tracker if their caregiver also discontinued use of their fitness tracker (95% CI 2.4-61.6). Compared with adolescents who discontinued use of the fitness tracker during the study, adolescents who continued to use the fitness tracker recorded a higher number of daily steps in months 2 and 3 of the study (mean 5760 vs 4148 in month 2, *P*=.005, and mean 5942 vs 3487 in month 3, *P*=.002).

**Conclusions:**

Despite high levels of satisfaction with the fitness trackers, fitness tracker discontinuation rates were high, especially among adolescents whose caregivers also discontinued use of their fitness tracker. More studies are needed to determine how to sustain the use of fitness trackers among adolescents with obesity and engage caregivers in adolescent weight management interventions.

## Introduction

Prevalence rates of obesity continue to increase among adolescents [1,2], translating into significant health consequences [3,4]. Expert guidelines recommend that pediatricians refer patients with obesity to tertiary care weight management clinics if they are unable to achieve a healthy weight in the primary care setting [5]. Patients who are adherent to interdisciplinary treatment in tertiary care weight management clinics demonstrate an improvement in weight status [6,7]. However, adherence to treatment is low, and attrition rates are high in tertiary care weight management clinics [8-10], especially among adolescents [11,12], thus limiting the number of patients who actually benefit from treatment. Indeed, tertiary care weight management clinics consistently report attrition rates >50% [8,9]. This underscores the need for innovative approaches to engage adolescents in weight management treatment.

One of the ways to engage patients in weight management treatment is to promote self-monitoring of lifestyle behaviors such as physical activity, which are associated with improved weight and health outcomes [13,14]. Self-monitoring is based on self-regulation theory, which proposes that monitoring one’s own behaviors leads to self-evaluation of how these behaviors impact progress toward a goal, which then positively reinforces behaviors that positively impact and negatively reinforces behaviors that negatively impact progress toward that goal [13,14]. Despite self-monitoring being a cornerstone of weight management treatment [15,16], it can be an arduous process [13,15,17]. Commercial wearable devices that track physical activity (fitness trackers) automate the self-monitoring process [18] and may be useful for engaging adolescents who are heavy technology users [19]. Fitness trackers have shown moderate success in assisting with weight management efforts in adults [20-23], but these studies have not extensively evaluated newer fitness trackers that are more accurate [24-27] and able to automate cognitive behavioral techniques such as goal setting and reinforcement [21].

Although there are many studies using research accelerometers with pediatric populations, commercial fitness trackers have not been extensively studied in the pediatric population. A recent systematic review found only 4 studies evaluating fitness trackers as an intervention among children, with only 1 of these studies evaluating fitness trackers as an intervention among adolescents [28]. These 4 pediatric studies reported positive effects of fitness trackers on physical activity but were limited by their use of older fitness tracker models and small sample sizes. In addition, none of these studies evaluated the use of fitness trackers in a clinical setting where physical activity data can be used to tailor counseling about physical activity for patients with obesity. Finally, none of these studies has evaluated the impact of providing caregivers with fitness trackers despite evidence that adolescent physical activity level is associated with the physical activity level of their caregivers [29-32]. Therefore, the purpose of this study was to test the use of a fitness tracker as an adjunct to family-based weight management treatment for adolescents. Our aim was to explore whether providing caregivers with a fitness tracker, in addition to providing adolescents with a fitness tracker, would improve an adolescent’s fitness tracker satisfaction, fitness tracker utilization, and physical activity levels.

## Methods

### Study Design

**Figure 1:**
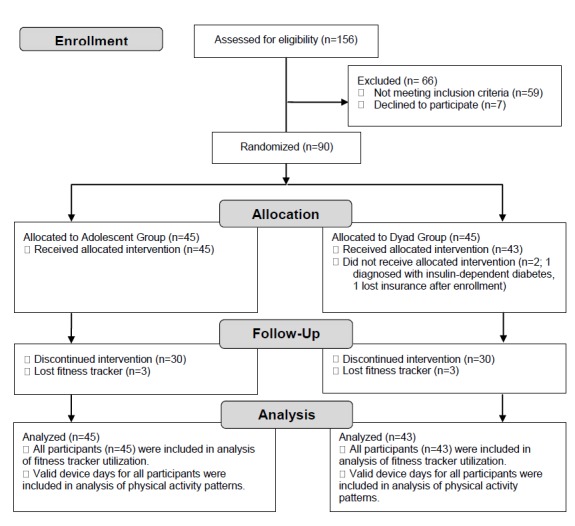
Consolidated Standards of Reporting Trials (CONSORT) diagram for pilot randomized trials.

A randomized trial of a fitness tracker was conducted over 3 months. Participants were randomized (1:1) using computer block randomization techniques into 1 of 2 groups: (1) adolescent group with the adolescent receiving a fitness tracker or (2) dyad group with the adolescent and their caregiver receiving a fitness tracker (Figure 1). Adolescents in both groups also received counseling regarding physical activity data as part of standard tertiary care weight management treatment, as described in detail below. At the time of trial implementation, the study did not meet requirements for registration on clinicaltrial.gov because of the sample size, lack of a control group naïve to an intervention, and intent to test the feasibility of the study procedures.

### Participants

Participants recruited for the trial were adolescents (aged 13-17 years) who were new patients to 1 of 2 tertiary care weight management clinics in a children’s health system. Patients were referred by their primary care provider to the clinic for having a body mass index percentile ≥85th percentile for age and having failed attempts at weight management in the primary care setting. One of the adolescent’s caregivers was also enrolled as a participant in the study. Participants were excluded if the adolescent had a primary genetic or endocrine syndrome associated with obesity or was taking a medication that would predispose them to weight gain; if the caregiver was not the adolescent’s legal guardian, did not reside in the same household, or was of limited English proficiency; or if either the adolescent or their caregiver was unable to understand how to use the fitness tracker, had used a fitness tracker before, did not have a smartphone or tablet computer, or had a condition limiting physical activity. Participants were also excluded from the study if they were unable to attend monthly weight management clinic visits for logistical or insurance reasons. This study was approved by the institutional review board, with both adolescents and their caregivers signing consent forms at the time of enrollment.

### Sample Size Derivation

The primary aim of this study was to assess the differential impact of an adolescent using the fitness tracker alone or in conjunction with their caregiver on physical activity patterns. As this is a novel comparison of the intervention, the effects are unknown. To detect a clinically meaningful difference in mean daily steps of 2000 (equating to approximately 1 mile of additional walking) between groups with a power of 80% and a level of significance of .05, assuming a 30% attrition rate for this 3-month study, we estimated that a sample size of 90 participants (45 in each experimental group) would be needed.

### Setting

Each of the 2 tertiary care weight management clinics was located inside a tertiary care children’s hospital. One clinic was located in the mid-Atlantic, and 1 clinic was located in the South Atlantic. Each clinic provides individualized, interdisciplinary weight management treatment to patients aged younger than 18 years (please see below section on Standard Weight Management Treatment for more details). Participants in the South Atlantic clinic were more likely to be of Hispanic background (48% [14/29] vs 14% [8/59] in the mid-Atlantic, *P*<.001), less likely to be of non-Hispanic white background (21% [6/29] vs 44% [26/59], *P*=.03), and more likely to have Medicaid insurance (55% [16/29] vs 29% [17/59], *P*=.02). There were no significant differences between participants at the 2 clinical sites in terms of gender, age, and weight status.

### Intervention

#### Standard Weight Management Treatment

All participants received the same standard weight management treatment, which was the typical routine care provided to all patients in the tertiary care weight management clinics. At their initial visit to the clinic, adolescents and their families underwent an assessment by a medical provider and received an individualized care plan. A typical plan included monthly clinic visits with an interdisciplinary team, which could include a medical provider, health educator, exercise physiologist, dietitian, or psychologist depending on identified needs. Care provided during each visit included management of medical comorbidities, nutrition education, physical activity counseling, and discussion of behavioral strategies such as goal setting and self-monitoring, with elements of motivational interviewing used to encourage behavior change. Care was targeted toward the entire family and stressed the importance of adoption of healthy lifestyle behaviors by the entire family. There was opportunity for the adolescent to participate in weekly personal training sessions at the mid-Atlantic site, which only 6 adolescents participated in.

#### Fitness Tracker Intervention

Adolescents in both the adolescent and dyad groups received a free fitness tracker. Caregivers of adolescents assigned to the dyad group also received a free fitness tracker. Participants were allowed to keep their fitness tracker as an incentive but were not provided additional incentives during the study to prevent influence on fitness tracker utilization patterns.

The fitness tracker used in this study was a slim, adjustable, water-resistant device worn on the wrist with a rubber wristband with a battery life of 5 days. The fitness tracker houses an accelerometer that tracks physical activity and sleep. This particular fitness tracker was found to be the second most accurate fitness tracker among 7 fitness trackers tested, with a reported error rate of 10% to 18% in measuring energy expenditure [25,33,34]. Each of 5 LED lights on the wristband illuminates with every 20% progress toward a daily activity goal set by the participants and vibrates when the daily goal is reached.

Data from the accelerometer wirelessly sync to computers and smartphones, with data viewable through a Web-based dashboard or mobile device app operated by the device manufacturer. These apps include features that allow participants to track physical activity (including steps taken, calories burned, and intensity of activity) and sleep (including duration of sleep and periods of restlessness) over different time intervals (daily, weekly, and monthly). Users are also able to earn virtual badges for reaching goals and compete in challenges with other fitness tracker users in real time through the Web-based app. Finally, participants can track food and water intake with the apps. Weekly reports are automatically emailed to users by the device manufacturer, containing a summary of their physical activity statistics, reminders about charging the fitness tracker, and links to healthy lifestyle resources.

At their initial visit to the weight management clinic, research staff assisted participants with installing the mobile app on their smartphone or tablet computer, setting up a fitness tracker account, and properly fitting the fitness tracker to each participant’s wrist. Research staff instructed participants on how to use the fitness tracker, including its different apps and functions, and how to sync the device. In addition, participants in the dyad group were encouraged to participate in fitness tracker challenges with one another. To account for variability in baseline physical activity, a phone call from research staff was made to adolescents 1 week after their initial visit to increase their step goal by 1000 steps above the average number of steps the adolescent had taken over the 3 days before the call. Research staff also provided assistance with fitness tracker issues during this call.

A secure research database was used to automatically and continuously collect coded data from the fitness tracker every time it was synced. Research staff texted generic reminders to participants to use, charge, and sync their fitness tracker on a weekly basis if they were noted to have zero steps recorded that week on the research database dashboard. During each weight management clinic visit, health care providers viewed the participant’s research database dashboard and reviewed their physical activity and progress for the past month, including amount of moderate to vigorous physical activity (MVPA) and step counts. Participants were encouraged to increase their MVPA and advised to strive for a new step goal that was 1000 steps above the average daily number of steps taken during their most consistent week.

### Measures

#### Adolescent Demographics

Adolescent age, sex, and insurance status were collected in the electronic health record as part of routine clinical care and entered into a REDCap database [35] by research staff at the initial visit. Adolescent race and ethnicity were reported via a REDCap questionnaire at the initial visit. Date of enrollment was also captured in REDCap and categorized according to season (summer being June to August, fall being September to November, winter being December to February, and spring being March to May).

#### Fitness Tracker Utility

A questionnaire was administered via REDCap survey to adolescents at each follow-up clinic visit (1, 2, and 3 months). Three items were asked to assess the adolescent’s perception about whether the fitness tracker was helping them meet their healthy lifestyle goals, be more physically active, and be more motivated to achieve a healthy weight. The adolescent was asked to rate how strongly they agreed with each statement on a 5-point Likert scale. Items were considered positively endorsed if they received a 4 or 5 on the Likert scale. Five additional items were asked to assess how frequently the adolescent used the functions of the fitness tracker (mobile app, Web-based dashboard, virtual rewards, challenges with their caregiver, and food and water log). Items responses were on a 5-point Likert scale. Items were considered frequently used (ie, used at least weekly) if they scored a 3, 4, or 5 on the Likert Scale.

#### Fitness Tracker Utilization and Physical Activity Patterns

Data on number of steps taken, number of calories burned, and number of minutes spent in MVPA were automatically and continuously collected by the fitness tracker and synced to the research database. Only physical activity on valid device days, which were defined as a day where 16 or more hours of nonzero steps were tracked, similar to criteria used in other studies [36,37], was used to the describe physical activity. For the purpose of analysis, physical activity data were described in terms of daily steps, daily minutes of MVPA, and daily calories burned. A mean daily value for each physical activity metric was calculated for each individual for each month of the study. Mean daily step data were further categorized by time of day (during school hours from 8 am to 4 pm or during nonschool hours from 4 pm to 11 pm) for some analyses. Adolescents were considered to have discontinued use of the fitness tracker if there was a time point after which they had no more valid device days, for at least 7 consecutive days.

### Data Analysis

Adolescent demographics were summarized and compared between the adolescent and dyad groups. To describe fitness tracker satisfaction, responses to the Fitness Tracker Utility questionnaire were summarized. Data collected from the fitness tracker were summarized to describe fitness tracker utilization and physical activity patterns. To explore the impact of providing caregivers with a fitness tracker as part of the intervention, intent-to-treat analysis was used to compare adolescent fitness tracker satisfaction, fitness tracker utilization, and physical activity patterns between the adolescent and dyad groups. Due to differences in season of enrollment and differences in demographics between the 2 clinical sites, we also tested to see if there were any differences in adolescent fitness tracker utility responses, fitness tracker utilization, and physical activity patterns between adolescents based on season of enrollment and clinical site. Due to high fitness tracker discontinuation rates, we also conducted exploratory analyses comparing demographic and physical activity patterns between adolescents who discontinued use of the fitness tracker during the study and those who did not. Chi-square or Fisher exact analyses were used to compare categorical variables, and 2-sample *t* tests were used to compare continuous variables. All tests were 2 tailed, with an overall level of significance of .05. Statistical software R was used.

## Results

### Adolescent Demographics

A total of 90 adolescents were recruited to receive a fitness tracker, with random assignment of 45 to the adolescent group and 45 to the dyad group (Figure 1). Two adolescents were excluded from the dyad group in the first month of the study because of insurance problems and diagnosis of insulin-dependent type 2 diabetes. The 88 remaining adolescents were representative of the patient population with the majority being female (61/88, 69%), having severe obesity (55/88, 63%), and representing a diversity of racial and ethnic backgrounds (non-Hispanic black [32/88, 36%], non-Hispanic white [26/88, 29.5%], and Hispanic [20/88, 23%]). There were no significant differences in baseline demographics between the adolescent and dyad groups (Table 1).

### Fitness Tracker Utility

Of the 72 adolescents who filled out the questionnaire at the 1-month follow-up visit, the majority reported that the fitness tracker was helping them meet their healthy lifestyle goals (50/72, 69%), be more physically active (49/72, 68%), and be more motivated to achieve a healthy weight (48/72, 66%). Of the 41 adolescents who completed the questionnaire at the 3-month follow-up visit, an even greater majority reported that the fitness tracker was helping them meet their healthy lifestyle goals (32/41, 78%), be more physically active (34/41, 82%), and be more motivated to achieve a healthy weight (33/41, 80%). Every month, more than 75% of adolescents reported accessing their mobile app weekly, but less than 45% of adolescents reported accessing their dashboard on the consumer website weekly. One-third of all adolescents used virtual rewards, and one-third of adolescents in the dyad group engaged in challenges with their caregiver. Food and activity logs were used rarely. There were no significant differences on any Fitness Tracker Utility questionnaire responses between the adolescent and dyad groups, between participants based on clinical site, or between participants based on the season of enrollment.

#### Fitness Tracker Utilization and Physical Activity Patterns

Table 2 describes fitness tracker utilization and physical activity patterns only on valid device days. Sixty-six (66/88, 68%) adolescents discontinued use of the fitness tracker before the end of the study, with 25 adolescents (25/88, 28%) discontinuing use of the fitness tracker in the first month. Notably, adolescents in the dyad group were 12.2 times more likely to discontinue using their fitness tracker if their caregiver also discontinued use of their fitness tracker (95% CI 2.4-61.6). Even among adolescents who continued to use the fitness tracker, the number of valid device days declined over time, from 29 days in the first month to 18 days in the third month of the study. Mean daily steps, minutes of MVPA, and calories burned on valid device days were consistently less than 8000 steps and 20 min of MVPA during each month. Number of steps per hour was significantly higher during school hours than during nonschool hours (484 vs 362 in the adolescent group and 473 vs 340 in the dyad group, *P*=.01).

There were no significant differences between the adolescent and dyad groups or between participants based on clinical site on any measures of fitness tracker utilization or physical activity patterns. However, participants who were enrolled in the summer burned a significantly higher number of calories in month 2 (mean 2549 vs 2241 among those enrolled in the fall and 2222 among those enrolled in the winter, *P*=.04) and month 3 (mean 2602 vs. 2191 among those enrolled in the fall and 2257 among those enrolled in the winter, *P*<.001).

**Table 1:** Adolescent demographics.

Adolescent demographics		Adolescent group^a^ (n=45)	Dyad group^b^ (n=43)	*P* value
Female, n (%)		31 (69)	30 (70)	1.00
**Race/ethnicity, n (%)**				.75
	Non-Hispanic black	18 (40)	14 (33)	
	Non-Hispanic white	12 (27)	14 (33)	
	Hispanic	11 (24)	9 (21)	
	Other	4 (9)	6 (14)	
Medicaid, n (%)		15 (33)	18 (42)	.55
Age (years), mean (SD)		15 (1.4)	14.7 (1.2)	.25
**Baseline weight category, n (%)**				.11
	Overweight or obese (BMI^c^ <99% for age)	21 (47)	12 (28)	
	Severe obesity (BMI ≥99% for age)	24 (53)	31 (72)	
**Clinic location, n (%)**				1.00
	Mid-Atlantic region	30 (67)	29 (67)	
	South Atlantic region	15 (33)	14 (33)	
**Season of enrollment, n (%)**				.62
	Summer (June to August)	17 (39)	20 (47)	
	Fall (September to November)	22 (50)	17 (40)	
	Winter (December to February)	5 (11)	6 (14)	

^a^Participants randomized to receive fitness tracker and counseling about physical activity data during standard weight management treatment.

^b^Participants randomized to receive fitness tracker and counseling about physical activity data during standard weight management treatment and for caregiver to also receive fitness tracker.

^c^BMI: body mass index.

**Table 2:** Adolescent fitness tracker utilization and physical activity patterns.

Fitness tracker utilization and physical activity patterns		Adolescent group^a^ (n=45)	Dyad group^b^ (n=43)	*P* value
**Discontinued using the fitness tracker,^c^ n (%)**				
	By 3 months	30 (67)	30 (70)	.93
	Month 1	14 (31)	11 (26)	.57
	Month 2	10 (22)	12 (28)	.53
	Month 3	6 (13)	7 (16)	.70
**Valid device days^d^ before device discontinuation, mean number of days (% per month)**				
	Month 1	29 (97)	28 (92)	.44
	Month 2	26 (86)	24 (81)	.43
	Month 3	19 (62)	17 (58)	.41
**Daily steps on valid device days^d^, mean (SD)**				
	Month 1	7541.1 (2891.2)	7356.3 (2611.4)	.75
	Month 2	7717.9 (3264.1)	7287.2 (2332.8)	.51
	Month 3	7873.7 (3035.3)	7150.4 (2543.4)	.31
	Weekdays	7859.1 (3002.2)	7676.2 (2431.1)	.75
	Weekends	6865.2 (3099.5)	6126.1 (2675.0)	.24
**Daily minutes of moderate to vigorous physical activity on valid device days^d^, mean (SD)**				
	Month 1	20.7 (34.0)	17.5 (29.8)	.64
	Month 2	15.3 (29.9)	13.6 (26.1)	.78
	Month 3	17.7 (33.1)	12.2 (24.7)	.38
**Daily calories burned on valid device days^d^, mean (SD)**				
	Month 1	2665.9 (500.5)	2670.3 (477.4)	.97
	Month 2	2700.7 (553.0)	2666.6 (514.8)	.78
	Month 3	2706.0 (554.2)	2653.4 (529.3)	.70
**Hourly steps on valid device days^d^, mean (SD)**				
	School hours (8 am to 4 pm)	483.5 (187.4)	473.9 (145.2)	.79
	Nonschool hours (4 pm to 11 pm)	361.6 (174.7)	340.1 (162.3)	.55

^a^Participants randomized to receive fitness tracker and counseling about physical activity data during standard weight management treatment.

^b^Participants randomized to receive fitness tracker and counseling about physical activity data during standard weight management treatment and for caregiver to also receive fitness tracker.

^c^Fitness tracker considered to be discontinued if there was a time point before the last week of the study after which there were 0 valid device days.

^d^A day was considered to be nonvalid if there were 16 or more hours of 0 steps.

#### Comparison of Adolescents Based on Fitness Tracker Discontinuation

Table 3 compares demographic characteristics and physical activity patterns between adolescents who discontinued use of the fitness tracker during the study and adolescents who continued to use the fitness tracker for the duration of the study. Adolescents who continued to use the fitness tracker were older (mean age 15.3 vs 14.6 years, *P*=.02) and were more likely to have been enrolled in the study in the summer (*P*=.05). Adolescents who continued to use the fitness tracker recorded a significantly higher number of mean daily steps in months 2 and 3 of the study (5760 vs 4148 in month 2, *P*=.002, and 5942 vs 3487 in month 3, *P*=.002) and calories burned in month 3 of the study (2539 vs 2262, *P*=.03) on valid device days than adolescents who discontinued use of the fitness tracker during the study.

**Table 3:** Comparison of adolescents based on fitness tracker discontinuation.

Adolescent demographics		Discontinued fitness tracker^a^ (n=60)	Continued fitness tracker (n=28)	*P* value
Male, n (%)		20 (33)	7 (25)	.47
**Race/ethnicity, n (%)**				.39
	Non-Hispanic black	19 (32)	13 (46)	
	Hispanic	16 (27)	4 (14)	
	Non-Hispanic white	19 (32)	7 (25)	
	Other	6 (10)	4 (14)	
Medicaid, n (%)		26 (43)	7 (25)	.16
Age (years), mean (SD)		14.6 (1.3)	15.3 (1.2)	.02
**Baseline weight category, n (%)**				1.00
	Overweight/obese (BMI^b^ <99% for age)	23 (38)	10 (36)	
	Severe obesity (BMI ≥99% for age)	37 (62)	18 (64)	
**Clinic location, n (%)**				.40
	Mid-Atlantic region	38 (63)	21 (75)	
	South Atlantic region	22 (37)	7 (25)	
**Season of enrollment, n (%)**				.05
	Summer (June to August)	23 (38)	14 (50)	
	Fall (September to November)	32 (53)	8 (29)	
	Winter (December to February)	5 (8)	6 (21)	
**Daily steps on valid device days^c^, mean (SD)**				
	Month 1	5768.0 (3027.4)	6096.0 (2421.3)	.62
	Month 2	4147.6 (3133.3)	5760.4 (1902.1)	.005
	Month 3	3486.5 (3111.3)	5942.0 (2360.1)	.002
**Daily minutes of moderate to vigorous physical activity on valid device days^c^, mean (SD)**				
	Month 1	20.6 (33.3)	16.4 (29.4)	.56
	Month 2	14.7 (30.8)	14.2 (23.5)	.93
	Month 3	15.8 (32.4)	13.2 (25.9)	.71
**Daily calories burned on valid device days^c^, mean (SD)**				
	Month 1	2469.8 (544.7)	2568.5 (472.4)	.41
	Month 2	2279.2 (606.6)	2525.0 (404.5)	.16
	Month 3	2261.8 (545.5)	2538.9 (428.7)	.03

^a^Fitness tracker considered to be discontinued if there was a time point before the last week of the study after which there were 0 valid device days.

^b^BMI: body mass index.

^c^A day was considered to be nonvalid if there were 16 or more hours of 0 steps.

## Discussion

### Principal Findings

Our study is the first to describe the use of a fitness tracker as an adjunct to family-based weight management treatment among a cohort of adolescents with obesity, a historically difficult to engage patient population with lower levels of physical activity and worse health consequences, and to describe the impact of providing fitness trackers to caregivers in support of their adolescents. Although the majority of adolescents in this study reported that the fitness tracker was helpful in their weight management efforts, discontinuation rates were high. In addition, there was no impact of providing caregivers with fitness trackers on adolescent physical activity levels; caregiver fitness tracker discontinuation rates were also high and associated with adolescent fitness tracker discontinuation. Adolescents who did not discontinue use of the fitness tracker by the end of the study were able to maintain physical activity levels, whereas those who discontinued use of their fitness tracker during the study had a decline in physical activity levels over time.

The majority of adolescents in this study reported that the fitness tracker was helpful in their weight management efforts and reported accessing their physical activity data weekly through a mobile app, supporting the utility of physical activity feedback especially when easily accessible via mobile technology. This is consistent with other studies that have demonstrated high levels of acceptability of fitness trackers among children and adolescents [38-40]. For example, the majority of adolescents in a study by Slootmaker et al found value in the physical activity assessments provided by fitness trackers [40]. In addition, qualitative studies by Schaefer et al found that pre-adolescents enjoyed the feedback feature of fitness trackers and that these features promoted self-reflection about their physical activity [38,39].

Despite high acceptability rates, the majority of adolescents discontinued use of the fitness tracker by the end of the study. Even among adolescents who continued to use the device for the entire study, the number of days the fitness tracker was used declined each month. Adherence to fitness trackers and discontinuation of fitness trackers have also been a major challenge in other studies [28,38,40,41]. A systematic review of randomized trials using accelerometers to measure physical activity in children found that one-third of children were not adherent to using accelerometers. In trials of commercial fitness trackers as the actual intervention, adherence is even worse. For example, only 24% of adolescents in Slootmaker et al’s study provided fitness tracker data for the entire study [40], and among pre-adolescents in Schaefer et al’s study, there was a 50% decrease in the use of the fitness tracker in the first month [38].

There may be several reasons for fitness tracker discontinuation over time. Seasonality appears to play a role, with participants who enrolled in the summer being more likely to continue use of their fitness tracker. This is likely because adolescents have more time when not in school, and the weather is more conducive in the summer for engaging in physical activity and use of the fitness tracker. There may also be other reasons that are more psychological in nature. For example, a recent mixed-methods study by Kerner and Goodyear found that adolescents lost motivation to use a fitness tracker over time because of feelings of incompetence when not achieving their goal [42], and a review by Sullivan and Lachman noted that inactive individuals struggle to come up with a plan for how to be physically active and overcome obstacles to being physically active [43], barriers that are not addressed with current fitness trackers.

In yet another study, Schaefer et al noted that utilization of the fitness tracker was highest during times where there was contact with research staff, suggesting that in-person support may still be a necessary component to engagement despite the automatic support inherent in the fitness tracker itself [38]. Similarly, utilization of the fitness tracker was highest in our study during the first month when contact with research staff was more frequent. At the same time, only about one-third of adolescents in our study used the automated functions of the fitness tracker that provide positive reinforcement such as rewards and challenges, which are important for behavior change, especially among inactive individuals [43], suggesting that more directive guidance on how to use these functions may also help to increase utilization of the fitness tracker and promote physical activity.

Fitness tracker discontinuation rates of adolescents in the dyad group were strongly associated with those of caregivers in the dyad group, suggesting that there is a strong influence of caregiver actions on adolescents. This is not surprising, given the importance of caregivers as role models to their children and the importance of the home environment to a child’s lifestyle habits [29-32]. However, caregivers in the dyad group also demonstrated low fitness tracker utilization rates, which may explain why adolescents in the dyad group did not demonstrate better fitness tracker utilization and physical activity patterns than adolescents in the adolescent group. Indeed, these findings suggest that more attention to increasing the physical activity of caregivers may be necessary to indirectly promoting physical activity in their adolescents.

At the same time, the lack of difference in outcomes between the adolescents in the adolescent and dyad groups may also speak to the need to focus efforts on peer-based interventions, especially among this age group that is more responsive to peer influence and support [44-46]. For example, Schaefer et al found that pre-adolescents in their study engaged with their fitness tracker most when competing with peers [38]. This concept is supported by studies demonstrating the positive effect of exergaming on physical activity among children, especially when peer socialization elements are incorporated [47-49]. Social media trends such as Pokemon GO further exemplify the role of social gaming in motivating individuals to be physically active [50,51]. Incorporating peer socialization as a component of fitness tracker interventions may also enhance physical activity outside of the school setting, which is important given that our study and others have observed significantly lower physical activity levels after school hours than during school hours [52].

Physical activity levels did not improve over the course of the study and were lower than evidence-based recommendations of 10,000 steps or 60 min of MVPA every day [53]. This lack of improvement in physical activity is in contrast to other studies reporting positive effects of fitness trackers on physical activity levels [20,28] but is consistent with studies reporting generally low levels of MVPA among children in this country, especially those with obesity and of adolescent age, who are a very difficult group to motivate to be more active [52,54,55] for many of the reasons that Sullivan and Lachman note in their study on inactive adults [43]. Notably, adolescents who continued to use the fitness tracker for the entire study period maintained their physical activity levels, whereas adolescents who discontinued use of the fitness tracker by the end of the study demonstrated a decline in their physical activity levels over the course of the study. This suggests that fitness tracker use may at least promote maintenance of physical activity levels among adolescents, even if it does not encourage an increase in physical activity levels. Because higher physical activity levels are associated with long-term weight loss and health benefits [12,13,56], this finding supports the importance of using fitness trackers with adolescents who are most likely to benefit from them or finding ways to increase adolescent engagement with fitness trackers.

### Limitations

There were several limitations to this study. Although our study was larger than other fitness tracker trials in pediatric populations, the sample size was still small, and the duration of our study was short. As this was a pilot study, we also did not include a control group of adolescents receiving standard treatment in the weight management clinic. Because we wanted to evaluate a commercial fitness tracker as an adjunct to care, leveraging the automated functions of the fitness tracker, we provided minimal intervention or incentives beyond counseling around the fitness tracker data at routine clinic visits, and use of the intervention may have been limited by functions specific to the fitness tracker, including the need to sync and charge the device. Also, the majority of adolescents in our study reported that the fitness tracker was helpful, but this may have been subject to self-report bias. Collecting qualitative data about why adolescents discontinued use of their fitness tracker may have provided meaningful information to inform future interventions. In addition, fitness tracker discontinuation rates were high, limiting the data that were available for analysis of physical activity, potentially leading to under- or overestimation of physical activity among participants.

### Conclusions

Despite high levels of satisfaction with the fitness trackers among adolescents enrolled in a weight management clinic, fitness tracker discontinuation rates were high, suggesting that more guidance and support may be needed beyond what is provided by the fitness tracker alone. This includes the support of caregivers, among whom fitness tracker discontinuation rates were also high and associated with adolescent fitness tracker discontinuation. Adolescents who continued use of the fitness tracker for the entire study demonstrated sustained physical activity levels compared with the decline in physical activity levels seen among adolescents who discontinued use of the fitness tracker during the study, suggesting that fitness tracker use may promote maintenance of physical activity levels over time. We believe the findings of our study provide valid and valuable insight into how adolescents with obesity use fitness trackers and underscores the importance of continued research to identify which subgroups will benefit most from this type of intervention while identifying innovative ways to engage adolescents in use of fitness trackers over time, including enhanced caregiver or peer involvement and features that address barriers to physical activity specific to obese patients.

## Abbreviations

BMI: body mass index
MVPA: moderate to vigorous physical activity
